# Supplementary material for: Aberrant functional connectivity network in subjective memory complaint individuals relates to pathological biomarkers
Source: Transl Neurodegener. 2018 Oct 19;7:27. doi: 10.1186/s40035-018-0130-z (PMC6196458; doi:10.1186/s40035-018-0130-z)
Supplement: Supplementary file 1 — Flow chart of subjects inclusion. Details regarding CCI. Analysis based on extended CSF data. Analysis based on data after pre-whitening. Details regarding DC and EC calculation. Analysis based on the follow-up data. Head motion parameters of SMC individuals and NC. Results under different thresholds. (DOCX 1176 kb) [file 40035_2018_130_MOESM1_ESM.docx]

**Aberrant functional connectivity network in subjective memory complaint individuals relates to pathological biomarkers**

Kaicheng Li^1†^, Xiao Luo^1†^, Qingze Zeng^1^, Yeerfan Jiaerken^1^, Xiaojun Xu^1^, Peiyu Huang^1^, Zhujing Shen^1^, Jingjing Xu^1^, Chao Wang^1^, Jiong Zhou^2^, Minming Zhang^*1^, and for the Alzheimer’s Disease Neuroimaging Initiative^3^

^1^Department of Radiology, 2nd Affiliated Hospital of Zhejiang University School of Medicine, China

^2^Department of Neurology, 2nd Affiliated Hospital of Zhejiang University School of Medicine, China

^3^Data used in the preparation of this article were obtained from the Alzheimer’s disease Neuroimaging Initiative (ADNI) database (http://www.adni.loni.usc.edu). As such, the investigators within the ADNI contributed to the design and implementation of ADNI and provided data but did not participate in analysis or writing of this report. A complete listing of ADNI investigators can be found at <http://adni.loni.usc.edu/wp-content/up> loads/how to apply/ADNI Acknowledgement List.pdf.

Corresponding to:

*Prof. Minming Zhang, MD, PhD

Department of Radiology, The 2nd Affiliated Hospital of Zhejiang University, School of Medicine, No.88 Jie-fang Road, Shang-cheng District, Hangzhou, China, 310009; Phone: 86-0571-87315255; Fax: 86-0571-87315255; Email address: [zhangminming@zju.edu.cn](mailto:zhangminming@zju.edu.cn)

Email address:

Kaicheng Li:15990004491@163.com

Xiao Luo: [luoxiao_zju@126.com](mailto:luoxiao_zju@126.com)

Qingze Zeng: 21718290@zju.edu.cn

Yeerfan Jiaerken: [11418165@zju.edu.cn](mailto:11418165@zju.edu.cn)

Xiaojun Xu: [xxjmailbox@163.com](mailto:xxjmailbox@163.com)

Peiyu Huang: [hpyzju@foxmail.com](mailto:hpyzju@foxmail.com)

Zhujing Shen: julywind0702@126.com

Jingjing Xu: [mcdxjj@163.com](mailto:mcdxjj@163.com)

Chao Wang: [wangchaosmart@zju.edu.cn](mailto:wangchaosmart@zju.edu.cn)

Jiong Zhou: yulanhuashu@vip.163.com

**Abstract**

**Background:** Individuals with subjective memory complaints (SMC) feature a higher risk of cognitive decline and clinical progression of Alzheimer’s disease (AD). However, the pathological mechanism underlying SMC remains unclear. We aimed to assess the intrinsic connectivity network and its relationship with AD-related pathologies in SMC individuals.

**Methods:** We included 44 SMC individuals and 40 normal controls who underwent both resting-state functional MRI and positron emission tomography (PET). Based on graph theory approaches, we detected local and global functional connectivity across the whole brain by using degree centrality (DC) and eigenvector centrality (EC) respectively. Additionally, we analyzed amyloid deposition and tauopathy via florbetapir-PET imaging and cerebrospinal fluid (CSF) data. The voxel-wise two-sample T-test analysis was used to examine between-group differences in the intrinsic functional network and cerebral amyloid deposition. Then, we correlated these network metrics with pathological results.

**Results:** The SMC individuals showed higher DC in the bilateral hippocampus (HP) and left fusiform gyrus and lower DC in the inferior parietal region than controls. Across all subjects, the DC of the bilateral HP and left fusiform gyrus was positively associated with total tau and phosphorylated tau_181_. However, no significant between-group difference existed in EC and cerebral amyloid deposition.

**Conclusion:** We found impaired local, but not global, intrinsic connectivity networks in SMC individuals. Given the relationships between DC value and tau level, we hypothesized that functional changes in SMC individuals might relate to pathological biomarkers.

**Keywords:**

Subjective memory complaint; Functional connectivity; Graph theoretical analysis; Neuropathology; Eigenvector centrality; Degree centrality

**Introduction**

Subjective memory complaint (SMC) refers to self-perceived cognitive decline with normal objective cognitive performance [1]. Prior studies showed that SMC individuals might precede amnestic mild cognitive impairments (aMCI) and exhibit a high conversion risk of Alzheimer’s disease (AD) [2]. Moreover, longitudinal studies noted that the risk for SMC individuals to convert to MCI or AD is 4.5-6.5 times higher than healthy aging individuals [3-7]. Therefore, SMC might serve as the typical presymptomatic stage along the AD continuum [8].

Recent neuroimaging studies found that SMC individuals is accompanied by cortical atrophy [9, 10] and white matter (WM) abnormalities [11] in AD-related regions, such as the medial temporal lobe. Functionally, SMC individuals feature both functional connectivity and metabolic alterations in the medial temporal and occipitoparietal regions [12-16]. These results jointly suggested SMC as the middle stage between MCI and normal controls (NC) and demonstrated that SMC might be among the earliest AD clinical symptoms. Pathological changes may explain these neuroimaging abnormalities. For example, autopsy studies have found higher levels of amyloid-β deposits and tau tangles in SMC individuals than healthy aging [17]. Further, PET study found increased entorhinal cortical tauopathy in SMC individuals and noted that tauopathy might be the most suggestive sign of SMC [18]. Despite these findings, the link between AD-related biomarkers and functional changes in SMC individuals is unclear.

To cover this gap, we combined graph theoretical approaches based on resting-state functional MRI (rsfMRI) and pathological biomarkers. By definition, graph theoretical centrality considers the brain as one vast network and measures the overall importance of individual brain regions. In the present study, we assessed two representative centrality metrics, degree centrality (DC) and eigenvector centrality (EC), across the entire brain. These metrics could capture the functional relationships of a given voxel (node) within the entire connectivity matrix of the brain (connectome). Specifically, DC is a local metric, calculating the number of direct connections for a given node [19]. In other words, a higher DC represents more direct connections with the node. In contrast, EC is a global metric calculating both the number and the weight of the connections [20, 21]. A brain region with a higher EC value means strong connection with more nodes and with higher weighting (i.e., there is a central role for the region in the whole-brain connectome). Furthermore, we assessed amyloid deposition in a voxel-wise manner and explored pathological changes in SMC individuals. Additionally, we examined the possible amyloid burden, neuronal death, and accumulation of tangles based on cerebrospinal fluid (CSF) data [22] .

We aimed to explore the intrinsic functional network and its corresponding pathologies in SMC individuals. Based on previous studies, we hypothesized that SMC individuals had more severe topological network impairment and a higher pathological burden than controls, especially in regions susceptible to AD pathologies such as the temporal and parietal lobes [23]. Moreover, aberrant functional connectivity metrics might relate to pathological change.

**Methods and Materials**

***Alzheimer’s disease neuroimaging and initiative***

Data used in the preparation of this article were obtained from the Alzheimer’s Disease Neuroimaging Initiative (ADNI) database (http://adni.loni.usc.edu). The ADNI was initially launched in 2004 (ADNI-1), and additional recruitment was made through ADNI-GO in 2009, ADNI-2 in 2010 and ADNI-3 in 2016. The primary goal of the ADNI has been to identify serial MRI, PET, biomarkers and genetic characteristics that would support the early detection and tracking of AD, and improve clinical trial design. For up-to-date information, see <http://www.adni-info.org>.

***Study participants***

This study was approved by the Institutional Review Boards of all participating institutions, and informed written consent was obtained from all participants at each site. We included 44 SMC individuals and 40 well-matched normal controls (NC) from the ADNI database (Supplementary Material 1). All participants underwent structural scans, rsfMRI scans, florbetapir PET amyloid scans, and comprehensive neuropsychological assessments at the same time point. The inclusion criteria for NC included the following: (a) having an Mini-Mental State Examination (MMSE) score between 24 and 30; (b) having a clinical dementia rating (CDR) score of 0; (c) having a normal Wechsler Memory Scale Logical Memory, WMS-LM, delay recall performance (in detail: ≥ 9 for subjects with 16 or more years of education; ≥ 5 for subjects with 8–15 years of education; and ≥ 3 for 0–7 years of education); (d) non-clinical depression (geriatric depression scale-15, GDS-15 score < 6) [24]; and (e) non-demented.

The inclusion criteria for SMC individuals included the following: (a) having a self-reported persistent memory decline assessed by using the Cognitive Change Index (CCI; the total score from the first 12 items ≥ 16, Supplementary Material 2) [10]; (b) having a normal cognitive performance (as for memory: having a normal WMS-LM delay recall performance; as for general mental status: having a normal MMSE (between 24 and 30) and a CDR score of 0) [1].

We excluded subjects with the following manifestations: (a) significant medical, neurological, and psychiatric illness; (b) obvious head trauma history; (c) use of non-AD-related medications known to influence cerebral function; (d) clinical depression; (e) alcohol or drug abuse; (f) left-handedness. After careful screening, we excluded 14 SMC individuals (three subjects with abnormal cognitive abilities, three subjects scanned with different rsfMRI acquisition parameters, six subjects with amyloid-PET data missing, two subjects with excessive head motion, Supplementary Material 1) **Table 1** shows the demographics of the included 44 SMC subjects and 40 well-matched NC subjects.

***Neuropsychological and CSF data acquisition***

All subjects underwent comprehensive neuropsychological tests, including assessment of general mental status (Mini-Mental State Examination, MMSE) and other cognitive domains, involving memory function (Auditory Verbal Learning Test, AVLT; WMS-LM, immediate and delayed memory), attention (Trail-Making Test part A, TMT-A), visuospatial function (Clock-Drawing Test, CDT), executive function (Trail-Making Test part B, TMT-B), and language ability (Boston Naming Test, BNT). Moreover, we also used Everyday Cognition (Ecog, Participant version and Informant version) to assess the subjective and partner-based cognitive complaints.

CSF biomarkers included amyloid-beta 1–42 (Aβ_1-42_), total tau (t-tau), and phosphorylated tau at position 181 (p-tau_181_), measured by the fully automated Roche Elecsys and Cobas e immunoassay analyzer system as previously described [25]. Notably, not all subjects had CSF sample since lumbar puncture is an invasive procedure. To ensure that pathology biomarkers accurately reflected the functional profile, we only included CSF samples at the same time as the rsfMRI acquisition (Supplementary Material 3). Thus, 19 out of 44 SMC individuals and 28 out of 40 NC had CSF samples available.

***MRI acquisition and pre-processing***

We acquired the T1-weighted images using the following parameters: repetition time (TR)=2300 ms; echo time (TE)=2.98 ms; inversion time (TI)=900 ms; 170 sagittal slices; within plane FOV=256 × 240 mm^2^; voxel size=1.1 × 1.1×1.2 mm^3^; flip angle=9°; bandwidth=240 Hz/pix. The rsfMRI images were obtained using an echo-planar imaging sequence with the following parameters: TR=3000 ms; TE=30 ms; the number of slices=48; slice thickness=3.3 mm; spatial resolution=3.31×3.31×3.31 mm^3^. According to the scan protocol, all subjects were instructed to open their eyes and keep at rest calmly during the scan.

We pre-processed all neuroimaging data using the Data Processing Assistant and Resting-State FMRI (DPARSF; www.[rfmri.org/DPASFA](http://rfmri.org/DPASFA)) [26] based on the platform of Statistical Parametric Mapping 8 (SPM8; www.fil.ion.ucl.ac.uk/spm) [27]. First, we discarded the first ten image volumes of rsfMRI scans for the signal equilibrium and subject’s adaptation to the scanning noise. Then, we corrected the remaining 130 images for timing differences and head motion [28]. Here, we discarded the image data with more than 2.5 mm maximum displacement in any of the x, y, or z directions or 2.5° of any angular motion. Subsequently, based on rigid-body transformation, we co-registered T1-weighted images to the mean rsfMRI image and spatially normalized these images to the Montreal Neurological Institute (MNI) standard space. The standardized image was subsequently re-sampled into 3 mm × 3 mm × 3 mm cubic voxel. Then, we performed a detrend and filter procedure (0.01 Hz < f < 0.08 Hz) to remove the bias from the high-frequency physiological noise and the low-frequency drift. Finally, we scrubbed the data to reduce motion-related artifacts by using a framewise displacement (FD) threshold of 0.5, deleting one time point before and two time points after “bad” time points [29]. To remove residual effects of motion and other non-neuronal factors, we corrected covariates including 24 head motion parameters and signals of white matter and CSF signal. Moreover, considering the possible effect of autocorrelation in fMRI time series, we additionally performed pre-whitening [30, 31] in the pre-processing by using FSL (Supplementary Material 4).

***Centrality metrics***

For each subject, we computed Pearson’s correlations between the time series of all pairs within the whole brain to produce the functional connectivity matrix. The procedure constrained by the gray matter mask generated by setting a threshold of 0.3 on the mean gray matter probability map. Then, we calculated the DC and EC metrics in a voxel-wise manner to quantify the local and global brain network integrity (Supplementary Material 5) [19]. In detail, we calculated DC by counting, for each voxel, the number of voxels it was connected to at a threshold of r ≥ 0.25. More details regarding DC processing are available in the literature [19, 32-35]. On the other hand, we calculated EC by counting the weighted number of correlations based on fast ECM (fECM) toolbox [32, 36, 37]. Then, all DC and EC maps underwent smoothing with full width at half maximum with a Gaussian kernel of 6 mm × 6 mm × 6 mm and Fisher’s Z transformation.

***PET acquisition and pre-processing***

We downloaded amyloid PET data from LONI in the most fully pre-processed format (series description in LONI Advanced Search: “AV45 Coreg, Avg, Std Img and Vox Siz, Uniform Resolution”). Subsequently, we coregistered the T1-weighted image to the mean amyloid PET image and spatially normalized these images to the Montreal Neurological Institute (MNI) space. A standardized image was subsequently re-sampled into 3 mm × 3 mm × 3 mm cubic voxel. Finally, each amyloid PET image was normalized to the whole cerebellum to create standardized uptake value ratio (SUVR) images.

***Statistical analyses***

We analyzed the demographic data using the chi-squared test for categorical data and t-test for continuous data (SPSS version 19.0). Then, we examined the neuroimaging metric differences (including DC, EC, and SUVR images) between the SMC and NC groups in a voxel-wise manner based on REST software (www.restfmri.net). In detail, we performed a two-sample t-test with age, gender, education, and GDS as the covariates, by setting the statistical threshold at P<0.001 and cluster size > ten voxels (uncorrected).

We defined regions showing significant differences between groups as the region of interest (ROIs) and extracted the mean features (DC, EC, and SUVR values) from them. Then, based on Spearman’s correlation, we correlated these neuroimaging metrics with neuropathological and neuropsychological results. To reduce the selection bias, we extended the CSF data and repeated the correlation analyses (Supplementary Material 3).

Moreover, to examine the stability of our results across time, we selected a subgroup of SMC individuals with both baseline and follow-up data from our original SMC subjects and repeated our analysis (Supplementary Material 6).

**Results**

***Demographic and neuropsychological data***

Descriptive data are presented as the mean ± standard deviation for continuous variables and percentage for dichotomous variables. The SMC individuals matched well with NC for age, gender, education, and APOE status. However, the SMC individuals showed higher GDS than the NC individuals. Regarding the cognitive performance and mean FD value (micromotion index), no significant difference existed between groups (Table 1, Supplementary Material 7). Moreover, SMC individuals had greater self-based/informant-based complaints than NC individuals in memory and global state.

***Centrality metrics***

The SMC individuals showed higher DC in the bilateral hippocampus (HP) and left fusiform gyrus and lower DC in the right inferior parietal region than NC individuals. However, no significant differences in EC existed between groups (Figure 1 and Table 2). Moreover, we adopted different statistical thresholds to explore the stability of our results (Supplementary Material 8).

***PET and CSF data***

The voxel-wise comparison of SUVR images showed no significant difference between the SMC and NC groups. No significant differences in CSF biomarkers existed between groups (Table 1).

***Correlation analyses***

Across groups, the DC value of bilateral HP and left fusiform gyrus was positively related with T-tau and P-tau_181_. Specifically, the DC value of the left HP was related to T-tau and P-tau_181_ (r=0.32, P＜0.05; r=0.37, P＜0.05, respectively); the DC value of the right HP was related to T-tau and P-tau_181_ (r=0.47, P＜0.05; r=0.45, P＜0.05, respectively); the DC value of the left fusiform gyrus was related to T-tau and P-tau_181_ (r=0.39, P＜0.05; r=0.40, P＜0.05, respectively) (Figure 2). More information is provided in Supplementary Material 3.

**Discussion**

Our study initially combined rsfMRI and pathological data to explore the intrinsic functional network and its possible pathological mechanism in SMC individuals. Based on centrality analyses, we found that the SMC individuals showed both impairment and compensation in the default mode network (DMN) at the local level (reflected by DC) but not at the global level (reflected by EC). Moreover, the links between the DC value and CSF tau level in the temporal regions suggested that the functional alternation in SMC individuals may result from tau-related pathologies.

SMC is at a stage of mild neuronal damage but still with sufficient functional compensation [38]. This stage may reflect the first effects of AD pathology on cognitive functioning between full compensation and the very first decline. Here, we found no difference in EC between groups, which suggested that SMC individuals have relatively intact global connectivity. This result was in line with the work of Wang et al. which reported similar global efficiency in SMC individuals and NC by examining the white matter connectivity network [39]. On the other hand, we found that the SMC individuals displayed increased DC in the medial temporal region (MTL, including the HP and fusiform gyrus) and decreased DC in the inferior parietal gyrus (IPG), suggesting aberrant local connectivity in the DMN. Another functional study came to similar conclusions, reporting that DMN function was alternated in SMC individuals and proposed it as the early AD-related connectivity failure [40]. Moreover, one study also reported reduced parietal activation while increased HP activation [41] in normal aging, demonstrating that successful memory encoding requires the coordination of neural activity in hippocampal and parietal regions. Accordingly, we hypothesized that reduced functional activity in the inferior parietal gyrus might indicate network deficiency, but increased activity in MTL might compensate for decreased memory in SMC individuals.

Supporting evidence for our hypothesis also comes from studies using different modalities. The inferior parietal region, as a functional core of the DMN, is vulnerable to functional connectivity breakdown in AD patients [33, 42]. Similar results can also be found in white matter network studies, demonstrating decreased nodal strength in the parietal region in SMC individuals [39, 43]. Moreover, early suffering from decreased glucose metabolic rates in the inferior parietal lobe in SMC individuals may help explain these connectivity abnormalities. [12]. Therefore, we proposed that the inferior parietal region is the primary target of functional decrease in SMC individuals which may further lead to cognition decline. Meanwhile, we observed that MTL exhibited increased function at the local level, which may help maintain cognitive performance in SMC individuals. Similarly, several memory encoding-related fMRI studies found increased activation in the MTL in SMC individuals [14, 15, 44], suggesting that this region may be involved in memory compensation [15]. Additionally, one white matter connectivity study reported higher nodal efficiency in MTL in SMC individuals [45]. Previous literature has hypothesized that before a global connectivity failure, brain regions with high activity could reflect an attempted compensation of early pathophysiological processes [46, 47]. Combined with the correlation between tau level and functional connectivity in the MTL, we proposed that the hyperconnectivity in SMC individuals is a result of brain plasticity after damage to the neural system. Conclusively, we hypothesized that both functional impairment and compensation simultaneously existed in SMC individuals, and such a functional pattern works jointly to maintain normal cognition in SMC individuals.

Regarding the pathological results, we did not observe significant amyloid differences between the SMC and NC groups. However, correlation analyses showed the links between MTL DC value and CSF tau level in all subjects. One possible explanation is that tau-mediated neuronal dysfunction [48, 49], but not amyloid burden is the initial pathology in SMC individuals. Some PET studies supported this interpretation to some extent [50-53]. Specifically, the SMC individuals tend to suffer tau pathology accumulation early in the MTL, in regions involved in memory function [18, 54-57]. In addition, Risacher et al. [58] reported that olfactory identification was more related to tauopathy than amyloid deposition in individuals with SMC. Another explanation is that the SMC group consists of a heterogeneous population [59]. Here, we included SMC individuals according to the framework and tried to meet the plus criteria [1]. Evidence such as informant-based complaints provides additional predictive ability for the progression to dementia in SMC individuals [60]. Notably, apart from AD-related pathologies, other neuropsychiatric factors such as depression or anxiety may also contribute to SMC [61-65]. These symptoms may also be manifestations of preclinical AD and can further lead to increases in amyloid formation and tau accumulation [66, 67] at the early stage of AD and could constitute a risk factor for subsequent dementia [68]. In our study, SMC individuals had a higher depression score (but within the clinically normal range), which was controlled to eliminate its possible effect according to the framework [1]. Considering the mixed function of subthreshold symptoms and pathologies, we still inferred that these possible mixed factors might affect the results to some extent.

There exist several limitations in our study. First, the sample size was relatively small, which reduced the statistical power. Future studies with larger sample sizes are required. Second, the SMC group is a heterogeneous group, easily resulted from other neuropsychological factors apart from AD-related pathologies. Future studies should consider these neuropsychiatric factors associated with SMC [59]. Moreover, the pathological ATN classification can help to define the SMC due to AD and should be used in the further analysis [69, 70]. Third, some CSF data are missing, which may lead to a selection bias. We performed a complementary correlation analysis based on extended CSF, which may support the stability of our findings to some extent. However, further studies with larger CSF sample sizes are urgent.

**Conclusion**

We found an impaired local, but not global, intrinsic functional network in SMC individuals, mainly involving the DMN. We hypothesized that the co-existence of functional impairment and compensation helped keep the normal cognitive in SMC individuals. Moreover, our results suggested that functional changes in SMC individuals may result from tau-related pathologies.

**Abbreviation:**

SMC: Subjective memory complaints

AD: Alzheimer’s disease

MCI: Mild cognitive impairments

NC: Normal control

PET: Positron emission tomography

rsfMRI: resting-state functional MRI

DC: Degree centrality

EC: Eigenvector centrality

HP: Hippocampus

CSF: Cerebrospinal fluid

WM: White matter

DMN: Default mode network

MTL: Medial temporal lobe

ADNI: Alzheimer’s disease Neuroimaging Initiative

CDR: Clinical dementia rating

WMS-LM: Wechsler Memory Scale Logical Memory

GDS: Geriatric depression scale

CCI: Cognitive Change Index

AVLT: Auditory Verbal Learning Test

TMT-A: Trail-Making Test, Part A

TMT-B: Trail-Making Test, Part B

CDT: Clock-Drawing Test

BNT: Boston Naming Test

Aβ1-42: amyloid-beta 1–42

T-tau: total tau

P-tau_181_ : hosphorylated tau at position 181

TR: epetition time

TE: echo time

TI: inversion time

MNI: Montreal Neurological Institute

FD: frame-wise displacement

fECM: Fast ECM

SUVR: standardized uptake value ratio

**Declarations**

***Ethics approval and consent to participate***

All procedures performed in studies involving human participants were in accordance by the ethical standards of the institutional and national research committee and with the 1964 Helsinki declaration and its later amendments or comparable ethical standards.
 Written informed consent was obtained from all participants and authorized representatives, and the study partners before any protocol-specific procedures were carried out in the ADNI study. More details in http://www.adni-info.org.

***Consent for publication***

Not applicable

***Availability of data and material***

The datasets generated and/or analysed during the current study are available in the ADNI study. More details in www.adni-info.org.

***Competing interests***

^†^ Kai-Cheng Li and Xiao Luo contributed equally to this work. The authors declare no conflict of interest.

***Funding***

This study was funded by National Key Research and Development Program of China (Grant No. 2016YFC1306600), Zhejiang Provincial Natural Science Foundation of China (Grant Nos. LZ14H180001 and Y16H090026), Young Research
Talents Fund, Chinese Medicine Science, and Technology Project of Zhejiang Province (Grant No. 2018ZQ035), the Fundamental Research Funds for the Central Universities (No.2017XZZX001-01), Zhejiang Medicine and Health Science and Technology Program (2018KY418).

***Authors' contributions***

KL and XL contributed equally to this work. KL designed the study and wrote the first draft of the manuscript. XL analysed the MRI data and wrote the protocol. JY modified the manuscript. QZ and JX collected the clinical and MRI data. JZ, CW, XX, PH, ZS and MZ assisted with study design and interpretation of findings. All authors have contributed to and approved the final manuscript.

All authors read and approved the final manuscript.

***Acknowledgements***

Data collection and sharing for this project was funded by the Alzheimer’s Disease Neuroimaging Initiative (ADNI) (National Institutes of Health Grant U01 AG024904) and DOD ADNI (Department of Defense award number W81XWH-12-2-0012). ADNI is funded by the National Institute on Aging, the National Institute of Biomedical Imaging and Bioengineering, and through generous contributions from the following: AbbVie, Alzheimer’s Association; Alzheimer’s Drug Discovery Foundation; Araclon Biotech; BioClinica, Inc.;Biogen; Bristol-Myers Squibb Company; CereSpir, Inc.; Eisai Inc.; ElanPharmaceuticals, Inc.; Eli Lilly and Company; EuroImmun; F. Hoffmann-La Roche Ltd. and its affiliated company Genentech, Inc.; Fujirebio; GE Healthcare; IXICO Ltd.; Janssen AlzheimerImmunotherapy Research & D development t , LLC.; J Johnson & Johnson PharmaceuticalResearch & Development LLC.; Lumosity; Lundbeck; Merck & Co., Inc.; MesoScaleDiagnostics, LLC.; NeuroRx Research; Neurotrack Technologies; Novartis PharmaceuticalsCorporation; Pfizer Inc.; Piramal Imaging; Servier; Takeda Pharmaceutical Company; andTransition Therapeutics. The Canadian Institutes of Health Research is providing funds to support ADNI clinical sites in Canada. Private sector contributions are facilitated by the foundation for the National Institutes of Health (www.fnih.org). The grantee organization is the Northern California Institute for Research and Education, and the study is coordinated by the Alzheimer’s Disease Cooperative Study at the University of California, San Diego.ADNI data are disseminated by the Laboratory for NeuroImaging at the University ofSouthern California

**Figure legend**

**Figure 1** shows the DC differences between SMC individuals and controls. SMC individuals showed higher DC (hot color) in the bilateral HP, left fusiform gyrus and lower DC (cold color) in the right inferior parietal region than controls (P<0.001, cluster size > 10 voxels, uncorrected, covariates including age, gender, education and geriatric depression scale).

**Abbreviations**: DC, degree centrality; SMC, subjective memory complaint; HP, hippocampus; IPG, inferior parietal region.

**Figure 2** shows the association between tau and DC value. Across groups, the DC value of the bilateral HP and left fusiform gyrus was positively associated with the T-tau and P-tau_181_ levels. **A:** DC value of left HP related to T-tau (r=0.32, P<0.05); **B:** DC value of left HP related to P-tau_181_ (r=0.37, P<0.05); **C:** DC value of right HP related to T-tau (r=0.47, P<0.05); **D:** DC value of right HP related to P-tau_181_ (r=0.45, P<0.05); **E:** DC value of left fusiform gyrus related to T-tau (r=0.39, P<0.05); **F:** the DC value of left fusiform gyrus related to P-tau_181_ (r=0.40, P<0.05). The scatter plot diagram displays the 95% confidence band of the best-fit line.

**Abbreviations:** DC, degree centrality; HP, hippocampus; T-tau, total tau; P-tau_181_, phosphorylated tau; the unit of CSF: (pg/ml).

**Reference**

[1] Jessen F Amariglio RE, van Boxtel M, Breteler M, Ceccaldi M, Chételat G, Dubois B, Dufouil C, Ellis KA, van der Flier WM, Glodzik L, van Harten AC, de Leon MJ, McHugh P. et al (2014) A conceptual framework for research on subjective cognitive decline in preclinical Alzheimer’s disease. *Alzheimers Dement* **10**, 844–852.

[2] Reisberg B., Prichep L., Mosconi L., John E. R., Glodzik-Sobanska L., Boksay I., Monteiro I., Torossian C., Vedvyas A., Ashraf N., Jamil I. A., de Leon M. J. (2008) The pre-mild cognitive impairment, subjective cognitive impairment stage of Alzheimer's disease. *Alzheimers Dement* **4**, S98-S108.

[3] Reisberg B Shulman MB, Torossian C, Leng L, Zhu W (2010) Outcome over seven years of healthy adults with and without subjective cognitive impairment. *Alzheimers Dement* **6**, 11–24.

[4] Jessen F., Wiese B., Bachmann C., Eifflaender-Gorfer S., Haller F., Kolsch H., Luck T., Mosch E., van den Bussche H., Wagner M., Wollny A., Zimmermann T., Pentzek M., Riedel-Heller S. G., Romberg H. P., Weyerer S., Kaduszkiewicz H., Maier W., Bickel H., German Study on Aging Cognition, Dementia in Primary Care Patients Study Group (2010) Prediction of dementia by subjective memory impairment: effects of severity and temporal association with cognitive impairment. *Arch Gen Psychiatry* **67**, 414-422.

[5] Jessen F Wolfsgruber S, Wiese B, et al. (2014) AD dementia risk in late MCI, in early MCI, and in subjective memory impairment. *Alzheimers Dement* **10**, 76–83.

[6] Kaup AR Nettiksimmons J, LeBlanc ES, Yaffe K (2015) Memory complaints and risk of cognitive impairment after nearly 2 decades among older women. *Neurology* **85**, 1852–1858.

[7] R€ onnlund M Sundstr€ om A, Adolfsson R, Nilsson LG (2015) Subjective memory impairment in older adults predicts future dementia independent of baseline memory performance: Evidence from the Betula prospective cohort study. *Alzheimers Dement* **11**, 1385–1392.

[8] Jessen F Amariglio RE, van Boxtel M, et al (2014) A conceptual framework for research on subjective cognitive decline in preclinical Alzheimer’s disease. *Alzheimers Dement* **10**, 844–852.

[9] Schultz SA Oh JM, Koscik RL, Dowling NM, Gallagher CL, Carlsson CM, Bendlin BB, LaRue A, Hermann BP, Rowley HA, Asthana S, Sager MA, Johnson SC, Okonkwo OC (2015) Subjective memory complaints, cortical thinning, and cognitive dysfunction in middle-aged adults at risk for AD. *Alzheimers Dement* **1**, 33-40.

[10] Saykin AJ Wishart HA, Rabin LA, Santulli RB, Flashman LA, West JD, McHugh TL, Mamourian AC (2006) Older adults with cognitive complaints show brain atrophy similar to that of amnestic MCI. *Neurology* **67**, 834-842.

[11] Li XY Tang ZC, Sun Y, Tian J, Liu ZY, Han Y (2016) White matter degeneration in subjective cognitive decline: a diffusion tensor imaging study. *Oncotarget* **7**, 54405-54414.

[12] Mosconi L De Santi S, Brys M, et al (2008) Hypometabolism and altered cerebrospinal fluid markers in normal apolipoprotein E E4 carriers with subjective memory complaints. *Biol Psychiatry* **63**, 609–618.

[13] Scheef L Spottke A, Daerr M, Joe A, Striepens N, K€ olsch H, et al (2012) Glucose metabolism, gray matter structure, and memory decline in subjective memory impairment. *Neurology* **79**, 1332–1339.

[14] Rodda J Dannhauser T, Cutinha DJ, Shergill SS, Walker Z (2011) Subjective cognitive impairment: Functional MRI during a divided attention task. *Eur Psychiatry* **26**, 457-462.

[15] Rodda JE Dannhauser TM, Cutinha DJ, Shergill SS, Walker Z (2009) Subjective cognitive impairment: increased prefrontal cortex activation compared to controls during an encoding task. *Int J Geriatr Psychiatry* **24**, 865-874.

[16] Sun Y Dai Z, Li Y, Sheng C, Li H, Wang X, Chen X, He Y, Han Y (2016) Subjective Cognitive Decline: Mapping Functional and Structural Brain Changes-A Combined Resting-State Functional and Structural MR Imaging Study. *Radiology* **281**, 185-192.

[17] Barnes L. L., Schneider J. A., Boyle P. A., Bienias J. L., Bennett D. A. (2006) Memory complaints are related to Alzheimer disease pathology in older persons. *Neurology* **67**, 1581-1585.

[18] Buckley R. F., Hanseeuw B., Schultz A. P., Vannini P., Aghjayan S. L., Properzi M. J., Jackson J. D., Mormino E. C., Rentz D. M., Sperling R. A., Johnson K. A., Amariglio R. E. (2017) Region-Specific Association of Subjective Cognitive Decline With Tauopathy Independent of Global beta-Amyloid Burden. *JAMA Neurol* **74**, 1455-1463.

[19] Zuo XN Ehmke R, Mennes M, Imperati D, Castellanos FX, Sporns O, Milham MP (2012) Network centrality in the human functional connectome. *Cereb Cortex* **22**, 1862-1875.

[20] Wink AM de Munck JC, van der Werf YD, van den Heuvel OA, Barkhof F (2012) Fast eigenvector centrality mapping of voxel-wise connectivity in functional magnetic resonance imaging: implementation, validation, and interpretation. *Brain Connect* **2**, 265–274.

[21] Binnewijzend MA1 Adriaanse SM, Van der Flier WM, Teunissen CE, de Munck JC, Stam CJ, Scheltens P, van Berckel BN, Barkhof F, Wink AM (2014) Brain network alterations in Alzheimer's disease measured by eigenvector centrality in fMRI are related to cognition and CSF biomarkers. *Hum Brain Mapp* **35**, 2383-2393.

[22] Blennow K., Hampel H. (2003) CSF markers for incipient Alzheimer's disease. *Lancet Neurol* **2**, 605-613.

[23] Frisoni GB Pievani M, Testa C, Sabattoli F, Bresciani L, Bonetti M, et al (2007) The topography of grey matter involvement in early and late onset Alzheimer's disease. *Brain* **130**, 720-730.

[24] Sheikh JI Yesavage JA (1986) Geriatric Depression Scale (GDS): recent evidence and development of a shorter version. *In: Brink TL, ed. Clinical Gerontology: A Guide to Assessment and Intervention. New York, NY: The Haworth Press;* , 165–173.

[25] Schindler S. E., Gray J. D., Gordon B. A., Xiong C., Batrla-Utermann R., Quan M., Wahl S., Benzinger T. L. S., Holtzman D. M., Morris J. C., Fagan A. M. (2018) Cerebrospinal fluid biomarkers measured by Elecsys assays compared to amyloid imaging. *Alzheimers Dement*.

[26] Chao-Gan Y Yu-Feng Z (2010) DPARSF: A MATLAB Toolbox for "Pipeline" Data Analysis of Resting-State fMRI. *Front Syst Neurosci* **4**, 13.

[27] Song XW Dong ZY, Long XY, Li SF, Zuo XN, Zhu CZ, He Y, Yan CG, Zang YF (2011) REST: a toolkit for resting-state functional magnetic resonance imaging data processing. *PLoS One* **6**, e25031.

[28] Friston K. J., Williams S., Howard R., Frackowiak R. S., Turner R. (1996) Movement-related effects in fMRI time-series. *Magn Reson Med* **35**, 346-355.

[29] Yan CG Cheung B, Kelly C, Colcombe S, Craddock RC, Di Martino A, Li Q, Zuo XN, Castellanos FX, Milham MP (2013) A comprehensive assessment of regional variation in the impact of head micromovements on functional connectomics. *Neuroimage* **76**, 183-201.

[30] Fiecas M., Cribben I., Bahktiari R., Cummine J. (2017) A variance components model for statistical inference on functional connectivity networks. *Neuroimage* **149**, 256-266.

[31] Arbabshirani M. R., Damaraju E., Phlypo R., Plis S., Allen E., Ma S., Mathalon D., Preda A., Vaidya J. G., Adali T., Calhoun V. D. (2014) Impact of autocorrelation on functional connectivity. *Neuroimage* **102 Pt 2**, 294-308.

[32] García-García I Jurado MÁ, Garolera M, Marqués-Iturria I, Horstmann A, Segura B, Pueyo R, Sender-Palacios MJ, Vernet-Vernet M, Villringer A, Junqué C, Margulies DS, Neumann J (2015) Functional network centrality in obesity: A resting-state and task fMRI study. *Psychiatry Research* **233**, 331-338.

[33] Buckner RL Sepulcre J, Talukdar T, Krienen FM, Liu H, Hedden T, Andrews-Hanna JR, Sperling RA, Johnson KA (2009) Cortical hubs revealed by intrinsic functional connectivity: mapping, assessment of stability, and relation to Alzheimer's disease. *J Neurosci* **29**, 1860-1873.

[34] Li S1 Ma X, Huang R, Li M, Tian J, Wen H, Lin C, Wang T, Zhan W, Fang J, Jiang G (2016) Abnormal degree centrality in neurologically asymptomatic patients with end-stage renal disease: A resting-state fMRI study. *Clin Neurophysiol* **127**, 602-609.

[35] Takeuchi H Taki Y, Nouchi R, Sekiguchi A, Hashizume H, Sassa Y, Kotozaki Y, Miyauchi CM, Yokoyama R, Iizuka K, Nakagawa S, Nagase T, Kunitoki K, Kawashima R (2015) Degree centrality and fractional amplitude of low-frequency oscillations associated with Stroop interference. *Neuroimage* **119**, 197-209.

[36] Wink AM de Munck JC, van der Werf YD, van den Heuvel OA, Barkhof F (2012) Fast eigenvector centrality mapping of voxel-wise connectivity in functionalmagnetic resonance imaging: Implementation, validation, and interpretation. *Brain Connect* **2**, 265-274.

[37] Lohmann G Margulies DS, Horstmann A, Pleger B, Lepsien J, Goldhahn D, Schloegl H, Stumvoll M, Villringer A,Turner R (2010) Eigenvector centrality mapping for analyzing connectivity patterns in fMRI data of the human brain. *PLoS One* **5**, e10232.

[38] Sperling R. A., Jack C. R., Jr., Aisen P. S. (2011) Testing the right target and right drug at the right stage. *Sci Transl Med* **3**, 111cm133.

[39] Wang XN Zeng Y, Chen GQ, Zhang YH, Li XY, Hao XY, Yu Y, Zhang M, Sheng C, Li YX, Sun Y, Li HY, Song Y, Li KC, Yan TY, Tang XY, Han Y (2016) Abnormal organization of white matter networks in patients with subjective cognitive decline and mild cognitive impairment. *Oncotarget* **7**, 48953-48962.

[40] Verfaillie S. C. J., Pichet Binette A., Vachon-Presseau E., Tabrizi S., Savard M., Bellec P., Ossenkoppele R., Scheltens P., van der Flier W. M., Breitner J. C. S., Villeneuve S., Group Prevent-Ad Research (2018) Subjective Cognitive Decline Is Associated With Altered Default Mode Network Connectivity in Individuals With a Family History of Alzheimer's Disease. *Biol Psychiatry Cogn Neurosci Neuroimaging* **3**, 463-472.

[41] Miller S. L., Celone K., DePeau K., Diamond E., Dickerson B. C., Rentz D., Pihlajamaki M., Sperling R. A. (2008) Age-related memory impairment associated with loss of parietal deactivation but preserved hippocampal activation. *Proc Natl Acad Sci U S A* **105**, 2181-2186.

[42] Buckner R. L., Andrews-Hanna J. R., Schacter D. L. (2008) The brain's default network: anatomy, function, and relevance to disease. *Ann N Y Acad Sci* **1124**, 1-38.

[43] Zhuang L., Sachdev P. S., Trollor J. N., Kochan N. A., Reppermund S., Brodaty H., Wen W. (2012) Microstructural white matter changes in cognitively normal individuals at risk of amnestic MCI. *Neurology* **79**, 748-754.

[44] Hafkemeijer A Altmann-Schneider I, Oleksik AM, van de Wiel L, Middelkoop HA, van Buchem MA, van der Grond J, Rombouts SA (2013) Increased Functional Connectivity and Brain Atrophy in Elderly with Subjective Memory Complaints. *Brain Connect* **3**, 353-362.

[45] Wang Y West J, Shen L, et al (2013) Altered connectome mapping in people with MCI and older adults with cognitive complaints. *Alzheimer’s Imaging Consortium Poster Presentations*, IC-P118.

[46] Elman J. A., Oh H., Madison C. M., Baker S. L., Vogel J. W., Marks S. M., Crowley S., O'Neil J. P., Jagust W. J. (2014) Neural compensation in older people with brain amyloid-beta deposition. *Nat Neurosci* **17**, 1316-1318.

[47] Hillary F. G., Grafman J. H. (2017) Injured Brains and Adaptive Networks: The Benefits and Costs of Hyperconnectivity. *Trends Cogn Sci* **21**, 385-401.

[48] Myeku N., Clelland C. L., Emrani S., Kukushkin N. V., Yu W. H., Goldberg A. L., Duff K. E. (2016) Tau-driven 26S proteasome impairment and cognitive dysfunction can be prevented early in disease by activating cAMP-PKA signaling. *Nat Med* **22**, 46-53.

[49] Ballatore C., Lee V. M., Trojanowski J. Q. (2007) Tau-mediated neurodegeneration in Alzheimer's disease and related disorders. *Nat Rev Neurosci* **8**, 663-672.

[50] Beckett LA Donohue MC, Wang C, Aisen P, Harvey DJ, Saito N; Alzheimer's Disease Neuroimaging Initiative (2015) The Alzheimer's Disease Neuroimaging Initiative phase 2: Increasing the length, breadth, and depth of our understanding. *Alzheimers Dement* **11**, 823-831.

[51] Rodda J., Okello A., Edison P., Dannhauser T., Brooks D. J., Walker Z. (2010) (11)C-PIB PET in subjective cognitive impairment. *Eur Psychiatry* **25**, 123-125.

[52] Chetelat G., Villemagne V. L., Bourgeat P., Pike K. E., Jones G., Ames D., Ellis K. A., Szoeke C., Martins R. N., O'Keefe G. J., Salvado O., Masters C. L., Rowe C. C., Australian Imaging Biomarkers, Lifestyle Research Group (2010) Relationship between atrophy and beta-amyloid deposition in Alzheimer disease. *Ann Neurol* **67**, 317-324.

[53] Ivanoiu A., Dricot L., Gilis N., Grandin C., Lhommel R., Quenon L., Hanseeuw B. (2015) Classification of non-demented patients attending a memory clinic using the new diagnostic criteria for Alzheimer's disease with disease-related biomarkers. *J Alzheimers Dis* **43**, 835-847.

[54] Schwarz A. J., Yu P., Miller B. B., Shcherbinin S., Dickson J., Navitsky M., Joshi A. D., Devous M. D., Sr., Mintun M. S. (2016) Regional profiles of the candidate tau PET ligand 18F-AV-1451 recapitulate key features of Braak histopathological stages. *Brain* **139**, 1539-1550.

[55] Braak H Braak E (1991) Neuropathological staging of Alzheimer-related changes. *Acta Neuropathologica* **82**, 239–259.

[56] Andrews-Hanna J. R., Reidler J. S., Sepulcre J., Poulin R., Buckner R. L. (2010) Functional-anatomic fractionation of the brain's default network. *Neuron* **65**, 550-562.

[57] Mormino E. C., Kluth J. T., Madison C. M., Rabinovici G. D., Baker S. L., Miller B. L., Koeppe R. A., Mathis C. A., Weiner M. W., Jagust W. J., Alzheimer's Disease Neuroimaging Initiative (2009) Episodic memory loss is related to hippocampal-mediated beta-amyloid deposition in elderly subjects. *Brain* **132**, 1310-1323.

[58] Risacher S. L., Tallman E. F., West J. D., Yoder K. K., Hutchins G. D., Fletcher J. W., Gao S., Kareken D. A., Farlow M. R., Apostolova L. G., Saykin A. J. (2017) Olfactory identification in subjective cognitive decline and mild cognitive impairment: Association with tau but not amyloid positron emission tomography. *Alzheimers Dement (Amst)* **9**, 57-66.

[59] Blackburn DJ Wakefield S, Shanks MF, Harkness K, Reuber M, Venneri A (2014) Memory difficulties are not always a sign of incipient dementia: a review of the possible causes of loss of memory efficiency. *Br Med Bull* **112**, 71–81.

[60] Caselli R. J., Chen K., Locke D. E., Lee W., Roontiva A., Bandy D., Fleisher A. S., Reiman E. M. (2014) Subjective cognitive decline: self and informant comparisons. *Alzheimers Dement* **10**, 93-98.

[61] Caracciolo B Gatz M, Xu W, Marengoni A, Pedersen NL, Fratiglioni L (2013) Relationship of subjective cognitive impairment and cognitive impairment no dementia to chronic disease and multimorbidity in a nation-wide twin study. *J Alzheimers Dis* **36**, 275-284.

[62] Comijs HC Deeg DJ, Dik MG, Twisk JW, Jonker C (2002) Memory complaints; the association with psycho-affective and health problems and the role of personality characteristics. A 6-year follow-up study. *J Affect Disord* **72**, 157-165.

[63] Steffens DC Potter GG (2008) Geriatric depression and cognitive impairment. *Psychol Med* **38**, 163-175.

[64] Derouesne C Lacomplez L, Thibault S, LePoncin M (1999) Memory complaints in young and elderly subjects. *Int J Geriatr Psychiat* **14**, 291–301.

[65] Jorm AF Butterworth P, Anstey KJ, et al (2004) Memory complaints in a community sample aged 60–64 years: associations with cognitive functioning, psychiatric symptoms, APOE genotype, hippocampus and amygdale volumes, and white matter hyperintensities. *Psychol Med* **34**, 1495–1506.

[66] Ramakers IH Verhey FR, Scheltens P, et al (2013) Anxiety is related to Alzheimer cerebrospinal fluid markers in subjects with mild cognitive impairment. *Psychol Med* **43**, 911–920.

[67] Green KN Billings LM, Roozendaal B, et al (2006) Glucocorticoids increase amyloid-beta and tau pathology in a mouse model of Alzheimer's disease. *The Journal of neuroscience : the official journal of the Society for Neuroscience* **26**, 9047–9056.

[68] Kaup AR Byers AL, Falvey C, Simonsick EM, Satterfield S, Ayonayon HN, et al (2016) Trajectories of Depressive Symptoms in Older Adults and Risk of Dementia. *JAMA Psychiatry* **73**, 525–531.

[69] Jack C. R., Jr., Bennett D. A., Blennow K., Carrillo M. C., Dunn B., Haeberlein S. B., Holtzman D. M., Jagust W., Jessen F., Karlawish J., Liu E., Molinuevo J. L., Montine T., Phelps C., Rankin K. P., Rowe C. C., Scheltens P., Siemers E., Snyder H. M., Sperling R., Contributors (2018) NIA-AA Research Framework: Toward a biological definition of Alzheimer's disease. *Alzheimers Dement* **14**, 535-562.

[70] Jack C. R., Jr., Knopman D. S., Chetelat G., Dickson D., Fagan A. M., Frisoni G. B., Jagust W., Mormino E. C., Petersen R. C., Sperling R. A., van der Flier W. M., Villemagne V. L., Visser P. J., Vos S. J. (2016) Suspected non-Alzheimer disease pathophysiology--concept and controversy. *Nat Rev Neurol* **12**, 117-124.

**Additional files:**

File name: additional file (contains supplementary material 1-8)

File format:pdf

Title of data:

supplementary material 1: Flow chart of subjects inclusion

supplementary material 2: Details regarding CCI

supplementary material 3: Analysis based on extended CSF data

supplementary material 4: Analysis based on data after pre-whitening

supplementary material 5: Details regarding DC and EC calculation.

supplementary material 6: Analysis based on the follow-up data

supplementary material 7: Head motion parameters of SMC individuals and NC

supplementary material 8: Results under different thresholds

**Table 1**

**The demographic, cognitive and neuropathological information**

| **Variables** | **NC** | **SMC** | **T/χ^2^- value** | **P value** |
| --- | --- | --- | --- | --- |
| **Number** | **40** | **44** |  |  |
| ***Demographic characteristics*** |  | | | |
| **Age,y, mean (SD)** | **75.10±5.39** | **73.78±5.81** | **1.08** | **0.28** |
| **Female, n(%)** | **22/18** | **24/20** | **0.002** | **0.57** |
| **Education (y), mean (SD)** | **16.70±2.39** | **16.66±2.60** | **0.08** | **0.94** |
| **Family,yes/no** | **23/17** | **14/30** | **7.28** | **0.03*** |
| **APOE** | **27/13** | **35/9** | **1.57** | **0.16** |
| ***CSF*** |  | | | |
| **Aβ_1–42_ (pg/ml)** | **1389.89±755.77** | **1552.79±683.30** | **-0.75** | **0.46** |
| **T-Tau(pg/ml)** | **235.32±83.46** | **259.52±68.76** | **-1.05** | **0.30** |
| **P-Tau_181_(pg/ml)** | **21.80±8.80** | **23.50±6.10** | **-0.73** | **0.47** |
| ***Neuropsychiatric Scores*** |  | | | |
| **CCI** |  | **31.37±8.30** |  | |
| **GDS** | **0.68±0.89** | **1.14±0.90** | **-2.36** | **0.02*** |
| ***General mental status*** |  | | | |
| **MMSE** | **29.05±1.18** | **29.36±0.75** | **-1.44** | **0.15** |
| ***Memory function*** |  | | | |
| **WMS-LM immediate** | **15.00±2.67** | **15.25±3.01** | **-0.39** | **0.70** |
| **WMS-LM delay** | **14.38±2.83** | **14.23±3.40** | **0.22** | **0.83** |
| **AVLT sum of trials 1–5** | **44.95±9.43** | **48.84±9.59** | **-1.86** | **0.07** |
| **AVLT30** | **7.18±4.14** | **8.88±4.18** | **-1.85** | **0.07** |
| ***Attention*** |  | | | |
| **Log-transformed TMT-A** | **1.50±0.12** | **1.48±0.14** | **0.59** | **0.55** |
| ***Decision-making function*** |  | | | |
| **Log-transformed TMT-B** | **1.79±0.16** | **1.84±0.17** | **-1.36** | **0.18** |
| ***Language*** |  | | | |
| **BNT total** | **28.94±1.05** | **28.52±1.50** | **1.14** | **0.26** |
| **Category fluency** | **21.75±4.06** | **22.20±5.02** | **-0.45** | **0.65** |
| ***Visuospatial processing*** |  | | | |
| **CDT** | **4.85±0.36** | **4.86±0.35** | **-0.18** | **0.86** |
| **Ecog PT: memory** | **1.53±0.41** | **1.94±0.60** | **-3.73** | **<0.001*** |
| **Ecog PT: global** | **1.35±0.26** | **1.55±0.38** | **-2.85** | **0.006*** |
| **Ecog Inf: memory** | **1.24±0.40** | **1.59±0.59** | **-3.18** | **0.002*** |
| **Ecog Inf: global** | **1.22±0.34** | **1.43±0.52** | **-2.15** | **0.03*** |

Data are presented as means ± standard deviations.

Abbreviation: SMC, Significant Memory Complaint; NC, Normal Controls; GDS, Geriatric Depression Scale; MMSE, Mini-Mental State Examination; WMS-LM, Wechsler Memory Scale Logical Memory; AVLT, Auditory Verbal Learning Test; TMT, Trail-Making Test; BNT, Boston Naming Test; CDT, Clock Drawing Test; E-Cog, Measurement of Everyday Cognition; PT, patient-based; Inf, Informant-based;

*p<0.05, significant difference between NC and SMC

Notably: The CSF data in Table 1 only represents the subjects who had CSF sample.

**Table 2**

**Results of degree centrality differences between SMC individuals and NC**

| **Brain regions** | **Cluter-size** | **Coordinates(MNI)** | | | **Peak intensity** |
| --- | --- | --- | --- | --- | --- |
|  |  | **X** | **Y** | **Z** |  |
| **R Hippocampus** | **23** | **27** | **-3** | **-24** | **3.89** |
| **L Hippocampus** | **11** | **-30** | **-18** | **-21** | **4.12** |
| **L Fusiform** | **16** | **-27** | **-45** | **-18** | **4.02** |
| **R Inferior parietal region** | **13** | **54** | **-21** | **27** | **-4.21** |
